# Supplementary material for: Correlation of Immune-Inflammatory Markers with Clinical Features and Novel Location-Specific Nomograms for Short-Term Outcomes in Patients with Intracerebral Hemorrhage
Source: Diagnostics (Basel). 2022 Mar 2;12(3):622. doi: 10.3390/diagnostics12030622 (PMC8947714; doi:10.3390/diagnostics12030622)
Supplement: Supplementary file 1 [file diagnostics-12-00622-s001.zip › diagnostics-1607970-supplementary.pdf]

Table S1. Univariate and multivariable analyses of clinical features and outcomes in 113 patients with thalamic hemorrhage

Characteristics	Univariate analysis						Stepwise logistic regression	
	Unfavorable outcome (mRS >3)			Death			Death	
	No (n = 32)	Yes (n = 81)	P value *	No (n =101)	Yes (n = 12)	P value *	OR (95% CI)	P value
Age (years)	62.8 (58.5-78.8)	67.0 (58.0-77.3)	0.568	64.1 (58.0-77.7)	70.0 (61.8-78.5)	0.323		
Hemoglobin (g/dL)	13.6 (12.6-15.6)	14.4 (12.8-15.2)	0.935	14.4 (12.8-15.4)	13.4 (12.8-14.6)	0.469		
Platelet (×10^9/L)	223 (177-243)	213 (163-270)	0.795	218 (171-265)	186 (160-250)	0.501		
WBC (×10^3/mL)	7.2 (5.8-8.8)	8.8 (6.6-11.5)	0.016	8.2 (6.2-10.1)	11.8 (8.4-14.2)	0.002	1.237 (0.813-1.882)	0.321
NC (×10^3/mL)	5.3 (3.8-6.8)	5.9 (4.4-8.7)	0.119	5.5 (4.2-7.1)	10.2 (6.3-11.7)	0.002		
NLR	3.4 (2.1-5.4)	3.4 (2.1-7.1)	0.511	3.2 (2.1-6.0)	7.6 (3.7-12.6)	0.006		
SII	709 (435-1156)	706 (477-1663)	0.556	670 (460-1098)	1688 (725-2400)	0.014		
Glucose (mg/dL)	118 (105-154)	140 (114-170)	0.111	125 (110-171)	144 (135-155)	0.131		
Creatinine (mg/dL)	1.0 (0.8-1.2)	1.0 (0.8-1.2)	0.577	1.0 (0.8-1.1)	1.2 (0.9-3.1)	0.066	1.465 (0.741-2.893)	0.272
Initial GCS score	15 (14.5-15.0)	13.0 (8.8-15.0)	<0.001	15.0 (12.8-15.0)	6.0 (4.0-8.5)	<0.001	0.881 (0.618-1.257)	0.186
ICH volume (cm^3)	2 (1-4)	5 (2-8)	<0.001	3 (1-5)	20 (16-35)	<0.001	1.618 (1.268-2.064)	<0.001
ICH score	0 (0-1)	1 (0-2)	<0.001	1 (0-1)	3 (2-4)	<0.001		
Presence of IVH	6 (19%)	43 (53%)	0.001	37 (37%)	12 (100%)	<0.001	-	0.997
Female gender	15 (47%)	29 (36%)	0.292	40 (40%)	4 (33%)	0.763		
Hypertension	28 (88%)	63 (78%)	0.299	81 (80%)	10 (83%)	>0.999		
Diabetes mellitus	8 (25%)	21 (26%)	>0.999	26 (26%)	3 (25%)	>0.999		
Heart disease	7 (22%)	9 (11%)	0.147	14 (14%)	2 (17%)	0.678		
Prior stroke	6 (19%)	18 (22%)	0.802	21 (21%)	3 (25%)	0.716		

Data are expressed as the median (1st -3rd quartile) or n (%);* Mann-Whitney test or Chi-square test. GCS, Glasgow Coma Scale; ICH, intracerebral hemorrhage; IVH, intraventricular hemorrhage; mRS, modified Rankin Scale; LOS, length of stay; NC, neutrophil count; NLR, neutrophil-to-lymphocyte ratio; SII, systemic immune-inflammation index; WBC, white blood cells.

Table S2. Univariate and multivariable analyses of clinical features and outcomes in 59 patients with pontine hemorrhage

Characteristics	Univariate analysis						Stepwise logistic regression	
	Unfavorable outcome (mRS >3)			Death			Death	
	No (n = 16)	Yes (n = 43)	P value *	No (n = 38)	Yes (n = 21)	P value *	OR (95% CI)	P value
Age (years)	61.9 (53.2-74.2)	55.6 (48.3-69.4)	0.298	57.7 (50.7-74.0)	55.6 (46.8-64.1)	0.419		
Hemoglobin (g/dL)	14.8 (13.9-15.8)	14.8 (12.0-16.7)	0.721	14.9 (12.9-16.6)	14.5 (12.3-16.2)	0.707		
Platelet ($\times10^9$/L)	216 (177-270)	211 (170-253)	0.664	213 (170-277)	211 (166-249)	0.719		
WBC ($\times10^3$/mL)	8.6 (6.3-10.2)	10.6 (8.2-13.3)	0.088	9.2 (6.4-11.5)	10.9 (9.2-14.6)	0.033	0.892 (0.696-1.142)	0.365
NC ($\times10^3$/mL)	6.4 (3.7-7.6)	6.8 (5.2-9.4)	0.380	6.0 (3.9-7.8)	7.0 (5.5-8.9)	0.141		
NLR	3.2 (1.9-4.6)	3.8 (1.7-6.0)	0.514	3.5 (1.9-5.0)	3.7 (1.5-6.3)	0.915		
SII	741 (478-112)	596 (278-1331)	0.903	647 (307-1175)	596 (264-1228)	0.701		
Glucose (mg/dL)	118 (98-147)	157 (139-224)	<0.001	131 (112-159)	165 (153-224)	<0.001	4.544 (0.200-103.103)	0.342
Creatinine (mg/dL)	1.0 (0.8-1.2)	1.1 (0.9-1.4)	0.181	1.0 (0.8-1.2)	1.1 (1.0-1.5)	0.119		
Initial GCS score	15 (15-15)	7 (3-12)	<0.001	15 (11-15)	3 (3-5)	<0.001	0.582 (0.449-1.754)	<0.001
ICH volume (cm^3)	1 (0-1)	4 (2-11)	<0.001	1 (0-2)	11 (5-16)	<0.001	1.202 (0.886-1.630)	0.137
ICH score	1 (1-1)	3 (2-3)	<0.001	1 (1-2)	3 (2-4)	<0.001		
Presence of IVH	0 (0%)	10 (23%)	0.487	2 (5%)	8 (38%)	0.003	6.514 (0.227-187.167)	0.274
Female gender	4 (25%)	14 (33%)	0.753	11 (29%)	7 (33%)	0.773		
Hypertension	12 (75%)	28 (65%)	0.545	27 (71%)	13 (62%)	0.564		
Diabetes mellitus	4 (25%)	8 (19%)	0.718	9 (24%)	3 (14%)	0.509		
Heart disease	2 (13%)	4 (9%)	0.658	3 (8%)	3(14%)	0.656		
Prior stroke	0 (0%)	6 (14%)	0.176	4 (11%)	2 (10%)	>0.999		

Data are expressed as the median (1st -3rd quartile) or n (%);* Mann-Whitney test or Chi-square test. GCS, Glasgow Coma Scale; ICH, intracerebral hemorrhage; IVH, intraventricular hemorrhage; mRS, modified Rankin Scale; LOS, length of stay; NC, neutrophil count; NLR, neutrophil-to-lymphocyte ratio; SII, systemic immune-inflammation index; WBC, white blood cells.

Table S3. Univariate and multivariable analyses of clinical features and outcomes in 48 patients with cerebellar hemorrhage

Characteristics	Univariate analysis						Stepwise logistic regression	
	Unfavorable outcome (mRS >3)			Death			Death	
	No (n = 16)	Yes (n = 32)	P value *	No (n = 39)	Yes (n = 9)	P value *	OR (95% CI)	P value
Age (years)	60.0 (56.5-64.5)	69.8 (63.4-79.5)	<0.001	63.7 (58.8-69.0)	73.0 (70.3-89.0)	0.007	1.083 (0.974-1.205)	0.093
Hemoglobin (g/dL)	14.1 (13.2-15.6)	13.8 (11.9-15.0)	0.341	14.1 (12.8-15.5)	12.3 (10.2-13.9)	0.116		
Platelet (×10^9/L)	217 (159-276)	198 (173-251)	0.645	200 (175-251)	195 (173-177)	0.910		
WBC (×10^3/mL)	9.8 (7.1-11.5)	11.9 (7.4-13.9)	0.143	10.6 (7.4-13.5)	7.6 (7.2-14.3)	0.937		
NC (×10^3/mL)	7.7 (4.4-9.4)	7.8 (4.8-12.0)	0.288	7.7 (5.1-11.0)	4.8 (4.4-9.8)	0.413		
NLR	5.0 (2.0-8.1)	4.1 (2.0-13.1)	0.718	5.1 (2.2-12.6)	2.2 (1.6-8.7)	0.245		
SII	828 (453-1650)	908 (303-3414)	0.793	897 (401-2588)	575 (278-2642)	0.348		
Glucose (mg/dL)	181 (141-228)	186 (156-239)	0.742	181 (151-237)	190 (166-238)	0.688		
Creatinine (mg/dL)	1.0 (0.9-1.7)	1.0 (0.8-1.5)	0.956	1.0 (0.8-1.6)	1.2 (0.9-1.4)	0.449		
Initial GCS score	15 (14-15)	11 (5-15)	0.002	15 (11-15)	3 (3-6)	<0.001	0.642 (0.493-0.836)	0.001
ICH volume (cm^3)	2 (1-7)	15 (6-33)	<0.001	6 (1-19)	24 (17-37)	0.003	1.032 (0.926-1.149)	0.370
ICH score	1 (1-1)	3 (1-4)	<0.001	1 (1-2)	4 (4-4)	<0.001		
Presence of IVH	3 (19%)	11 (34%)	0.328	9 (23%)	5 (55%)	0.099	0.439 (0.050-3.845)	0.457
Female gender	6 (38%)	12 (38%)	>0.999	15 (38%)	3 (33%)	>0.999		
Hypertension	10 (63%)	22 (69%)	0.750	26 (67%)	6 (67%)	>0.999		
Diabetes mellitus	4 (25%)	11 (34%)	0.742	13 (33%)	2 (22%)	0.699		
Heart disease	1 (6%)	7 (22%)	0.239	5 (13%)	3 (33%)	0.159		
Prior stroke	1 (6%)	7 (22%)	0.239	7 (18%)	1 (11%)	>0.999		

Data are expressed as the median (1st -3rd quartile) or n (%);* Mann-Whitney test or Chi-square test. GCS, Glasgow Coma Scale; ICH, intracerebral hemorrhage; IVH, intraventricular hemorrhage; mRS, modified Rankin Scale; LOS, length of stay; NC, neutrophil count; NLR, neutrophil-to-lymphocyte ratio; SII, systemic immune-inflammation index; WBC, white blood cells.

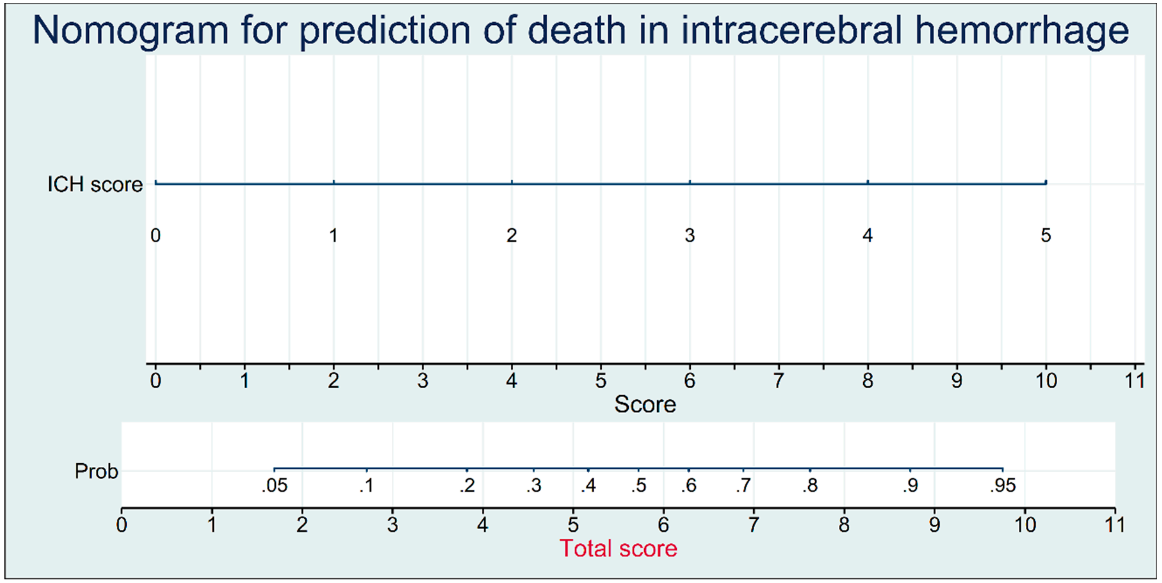

Figure S1. Nomogram for prediction of death due to all intracerebral hemorrhages using the intracerebral hemorrhage score system.
